# Intrinsically Stretchable and Healable Polymer Semiconductors

**DOI:** 10.1002/advs.202305800

**Published:** 2023-12-19

**Authors:** Xiang Xue, Cheng Li, Zhichun Shangguan, Chenying Gao, Kaiyuan Chenchai, Junchao Liao, Xisha Zhang, Guanxin Zhang, Deqing Zhang

**Affiliations:** ^1^ Beijing National Laboratory for Molecular Science CAS Key Laboratory for Organic Solids Institute of Chemistry Chinese Academy of Sciences Beijing 100190 China; ^2^ School of Chemical Sciences University of Chinese Academy of Sciences Beijing 100049 China

**Keywords:** dynamic bonding units, healable polymer semiconductors, intrinsically stretchable polymer semiconductors, polymer backbones, side chains

## Abstract

In recent decades, polymer semiconductors, extensively employed as charge transport layers in devices like organic field‐effect transistors (OFETs), have undergone thorough investigation due to their capacity for large‐area solution processing, making them promising for mass production. Research efforts have been twofold: enhancing the charge mobilities of polymer semiconductors and augmenting their mechanical properties to meet the demands of flexible devices. Significant progress has been made in both realms, propelling the practical application of polymer semiconductors in flexible electronics. However, integrating excellent semiconducting and mechanical properties into a single polymer still remains a significant challenge. This review intends to introduce the design strategies and discuss the properties of high‐charge mobility stretchable conjugated polymers. In addition, another key challenge faced in this cutting‐edge field is maintaining stable semiconducting performance during long‐term mechanical deformations. Therefore, this review also discusses the development of healable polymer semiconductors as a promising avenue to improve the lifetime of stretchable device. In conclusion, challenges and outline future research perspectives in this interdisciplinary field are highlighted.

## Graphical abstract

Intrinsically stretchable and healable polymer semiconductors hold great potential for flexible electronics. This review summarizes the key advancements of intrinsically stretchable and healable polymer semiconductors. The variation of charge transporting property and morphology evolution of these materials under stress and during healing process are discussed.

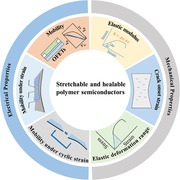

## 1 Introduction

The most significant advantage of polymer semiconductors over the inorganic counterparts is their intrinsic flexibility, stemming from the nature of molecular materials.^[^ ^1^, ^2^, ^3^, ^4^, ^5^, ^6^, ^7^, ^8^, ^9^ ^]^ Leveraging this unique advantage, polymer semiconductors are considered as ideal candidates for various flexible applications, including electronic paper, electronic skin, wearable electronics, and man–machine interface.^[^ ^10^, ^11^, ^12^, ^13^, ^14^, ^15^, ^16^, ^17^ ^]^ As the fundamental building blocks of flexible devices, stretchable organic field effect transistors comprise several components, encompassing flexible substrates, semiconducting layers, dielectric materials, and electrodes. All these elements must endure mechanical deformations and bending without compromising the electrical performance. Stretchable elastomers such as polydimethylsiloxane (PDMS) and polystyrene‐block‐poly(ethylene‐ran‐butylene)‐block‐polystyrene (SEBS) can be used as flexible substrates and serve as the foundation for the devices. Plastic materials such as poly(methyl methacrylate) (PMMA), which can function as dielectric layers, are typically flexible. Flexible electrodes based on either poly(3,4‐ethylenedioxythiophene) polystyrene sulfonate (PEDOT:PSS) or carbon nanotube (CNT) or silver nanowire (AgNW) networks have been investigated. Various strategies have been proposed for designing and preparing stretchable polymer semiconductors, which are composed of conjugated backbones and flexible side chains.^[^ ^16^, ^18^, ^19^, ^20^ ^]^ Ordinarily, the ordered packing of polymer chains is required for boosting the charge mobilities.^[^ ^2^ ^]^ But this will make the polymer semiconductors with high charge mobilities brittle. Thus, the poor mechanical property of polymer semiconductors is the short‐board of flexible devices.^[^ ^21^, ^22^, ^23^ ^]^

Specifically, polymer semiconductors consist of homo‐conjugated units or alternating electron‐donor (D) units/electron acceptor (A) units in the backbones and side alkyl chains. Planar and rigid building blocks such as diketopyrrolopyrrole (DPP),^[^ ^24^, ^25^, ^26^, ^27^, ^28^ ^]^ isoindigo (IID),^[^ ^29^, ^30^, ^31^, ^32^ ^]^ benzothiadiazole (BT),^[^ ^33^, ^34^, ^35^, ^36^, ^37^ ^]^ thienopyrroledione (TPD),^[^ ^38^, ^39^ ^]^ and naphthalenediimide (NDI)^[^ ^40^, ^41^, ^42^, ^43^ ^]^ have been extensively utilized to construct the conjugated backbones of polymer semiconductors. On the one hand, various methods have been devised to enhance interchain packing order and thin film crystallinity of polymer semiconductors, aiming to boost their charge mobilities.^[^ ^8^, ^9^, ^44^ ^]^ However, thin films of such polymer semiconductors, with good semiconducting performance, are brittle and stiff, demonstrating high tensile modulus up to several hundred MPa or even higher, which is likely to lead to device performance degradation under deformation.^[^ ^10^, ^16^, ^18^ ^]^ On the other hand, when thin film crystallinity of polymer semiconductors is reduced, and even thin films become amorphous, the tensile modulus can be significantly lowered and the resulting thin films become stretchable. Unfortunately, the charge transporting performance becomes inferior under those condition.^[^ ^45^, ^46^, ^47^ ^]^ Clearly, the structural requirements of charge mobility and stretchability of polymer semiconductors in the aggregate structures are mutually contradictory. Therefore, integrating excellent semiconducting and mechanical properties into a single conjugated polymer poses a significant challenge.

In recent years, several innovative strategies have been developed with the aim of achieving a trade‐off between charge transporting and stretchable properties. According to the way of strain energy dissipation,^[^ ^15^, ^16^ ^]^ the strategies can be divided into the following three categories. First, blending polymer semiconductors with insulated elastomers has emerged as a powerful way to improve the mechanical property of polymer semiconductors.^[^ ^48^, ^49^ ^]^ The conformational changes of the mobile polymer chains of the elastomers can effectively dissipate strain energy and maintain interconnected networks of conjugated polymers during deformation. Through optimization of the two‐component composite systems, nanoaggregates of polymer semiconductors are formed within the matrix of elastomers. Consequently, in some cases, even with high contents of insulating elastomers, the blending films show both high charge mobilities and excellent stretchable properties simultaneously.^[^ ^50^ ^]^ Second, geometry engineering has been employed to create microstructures such as curvilinear arrays and to fabricate stretchable polymer semiconductors with high charge mobilities.^[^ ^51^, ^52^, ^53^ ^]^ The strain energy can be dissipated through the deformation of microstructures. The difficulty of such strategy lies in how to achieve large‐scale and highly uniform microstructures. Third, through the engineering of the structures of conjugated backbones and side chains, numerous intrinsically stretchable polymer semiconductors have been reported.^[^ ^16^, ^18^ ^]^ Compared to the physical blending and geometry engineering methods, this represents a straightforward approach for stretchable polymer semiconductors, simplifying the device fabrication process. Consequently, intrinsically stretchable polymer semiconductors have received increasing attention recently.^[^ ^54^, ^55^, ^56^, ^57^ ^]^

In the following, we will discuss representative approaches for the construction of intrinsically stretchable polymer semiconductors. Emphasis will be placed on developing a profound understanding of the relationship among chemical structures of polymer semiconductors, their thin film structures, tensile modulus, strain energy dissipation, charge mobility, and morphology change before and after the application of strain. Additionally, we will discuss the research progresses on healable polymer semiconductors, which are useful to address the sustainability and longevity concerns associated with organic electronic devices. The fundamental understanding of the molecular structure‐morphology‐performance relationship for intrinsically stretchable and healable polymer semiconductors will be highlighted to guide future molecular designs. Finally, along with highlighting recent advances in this field, we will discuss existing challenges and potential opportunities.

## 2 Intrinsically Stretchable Polymer Semiconductors

**Figure 1:**
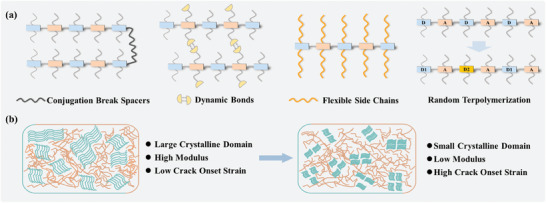
a) Illustration of the molecular design strategy for intrinsically stretchable polymer semiconductors, including the incorporation of CBSs, dynamic bonds, flexible side chains, and the third copolymerization component into the polymers. b) Illustration of the regulation of aggregation structures for intrinsically stretchable polymer semiconductors.

Intrinsically stretchable polymer semiconductors are referred to as a class of conjugated polymers exhibiting both semiconducting property and inherent stretchability without the need of special processing techniques. Unlike conventional rigid polymer semiconductors, intrinsically stretchable polymer semiconductors can undergo significant mechanical strain without compromising their semiconducting performances. To reduce the tensile modulus of conjugated polymers, several effective strategies have been proposed. These include the incorporation of conjugation break spacers (CBSs),^[^ ^45^, ^46^, ^47^, ^55^, ^58^, ^59^, ^60^, ^61^, ^62^, ^63^, ^64^, ^65^ ^]^ dynamic bonding units,^[^ ^66^, ^67^, ^68^, ^69^, ^70^, ^71^, ^72^, ^73^, ^74^, ^75^, ^76^, ^77^, ^78^ ^]^ flexible side chains^[^ ^56^, ^57^, ^79^, ^80^, ^81^, ^82^, ^83^, ^84^, ^85^, ^86^, ^87^, ^88^, ^89^ ^]^ and the third copolymerization component into polymer semiconductors (**Figure** **1**a).^[^ ^90^, ^91^, ^92^, ^93^ ^]^ The cornerstone of these strategies is precisely reducing the overall crystallinity of conjugated polymers without weakening the short‐range interchain packing order. In these cases, crystalline domains provide effective charge transport channels, while the amorphous domains are the main sites for strain energy dissipation and morphology evolution under deformation. Therefore, small crystalline domains distributed in amorphous domains have been considered as the ideal morphology for effective charge transporting and good stretchability (Figure 1b).^[^ ^16^ ^]^ In the following section, we will present recent key advancements in this area, with an emphasis on how the molecular structures affect their semiconducting and mechanical properties. **Table** **1** lists the molecular weights, tensile moduli, crack onset strains, charge mobilities of the intrinsically stretchable polymer semiconductors.

**Table 1:** Structural and performance parameters of stretchable polymer semiconductors.

Polymer	*M*_n_ [kDa]	PDI	Elastic modulus [MPa]	Crack onset strain	Initial mobility without strain [cm^2^ V^−1^ s^−1^]	Mobility under strain [cm^2^ V^−1^ s^−1^]	Mobility after cyclic strain [cm^2^ V^−1^ s^−1^]	Reference
P17^a)^	30.9	3.61	≈1100	25%	≈2.2	≈0.90 (*µ*_∥_) and ≈0.73 (*µ*_⊥_), at 100% strain	≈0.055 (*µ*_∥_) and ≈0.187 (*µ*_⊥_), 100 cycles, at 50% strain	[62]
P18^a)^	24.7	2.99	≈180	35%	≈1.7	≈0.92 (*µ*_∥_) and ≈0.56 (*µ*_⊥_), at 100% strain	N/A	[62]
P19^a)^	40.0	2.93	≈150	40%	≈2.2	≈0.92 (*µ*_∥_) and ≈0.74 (*µ*_⊥_), at 100% strain	N/A	[62]
P20^a)^	31.8	3.26	130	100%	≈1.3	≈1.0 (*µ*_∥_) and ≈0.6 (*µ*_⊥_), at 100% strain	≈0.351 (*µ*_∥_) and ≈0.559 (*µ*_⊥_), 100 cycles, at 50% strain	[62]
P20^b)^	31.8	3.26	130	100%	0.463	0.1 (*µ*_∥_) and ≈0.3 (*µ*_⊥_), at 100% strain	≈0.058 (*µ*_∥_) and 0.275 (*µ*_⊥_), 100 cycles, at 50% strain	[62]
P27^a)^	20.0	1.95	19	40%	4.08×10^−3^	3.4×10^−4^ (*µ*_∥_) and 3.6×10^−4^ (*µ*_⊥_), at 100% strain	1.25×10^−3^ (*µ*_∥_) and 1.85×10^−3^ (*µ*_⊥_), 100 cycles, at 60% strain	[58]
P28^a)^	17.4	1.61	N/A	40%	2.55×10^−3^	3.3×10^−4^ (*µ*_∥_) and 4.1×10^−4^ (*µ*_⊥_), at 100% strain	N/A	[58]
P29^a)^	26.3	1.78	49	20%	4.85×10^−3^	3.5×10^−4^ (*µ*_∥_) and 5.6×10^−4^ (*µ*_⊥_), at 100% strain	1.90×10^−3^ (*µ*_∥_) and 2.45×0^−3^ (*µ*_⊥_), 100 cycles, at 60% strain	[58]
P30^a)^	24.8	1.91	N/A	60%	2.38×10^−3^	2.7×10^−4^ (*µ*_∥_) and 4.3×10^−4^ (*µ*_⊥_), at 100% strain	N/A	[58]
P31^a)^	21.7	1.96	69	20%	2.69×10^−3^	2.5×10^−4^ (*µ*_∥_) and 3.3×10^−4^ (*µ*_⊥_), at 100% strain	5.84×10^−4^ (*µ*_∥_) and 7.80×10^−4^ (*µ*_⊥_), 100 cycles, at 60% strain	[58]
P32^a)^	14.9	1.55	N/A	>60%	1.28×10^−4^	2.0×10^−5^ (*µ*_∥_) and 3.4×10^−5^ (*µ*_⊥_), at 100% strain	N/A	[58]
P38^c)^	37.5	2.53	5.5	>85%	0.08	Maintain initial conductivity, at 100% strain	Maintain conductivity,1500 cycles, at 50% strain	[65]
P39^a)^	20.4	3.2	≈2200	≈5%	≈1.8	≈0.0008 (*µ*_∥_), at 50% strain ≈0.08 (*µ*_⊥_), at 75% strain	N/A	[76]
P41^a)^	16.2	3.0	≈340	≈120%	1.32	0.11 (*µ*_∥_) and 1.12 (*µ*_⊥_), at 100% strain	0.74 (*µ*_∥_) and 1.07 (*µ*_⊥_), 100 cycles, at 25% strain 0.017 (*µ*_∥_) and 0.977 (*µ*_⊥_), 100 cycles, at 100% strain	[76]
P41^b)^	16.2	3.0	≈340	≈120%	0.34	0.033 (*µ*_∥_) and 0.091 (*µ*_⊥_), at 100% strain	0.108 (*µ*_∥_) and 0.165 (*µ*_⊥_), 500 cycles, at 25% strain	[76]
P46^b)^	23.6	4.2	≈435	40%	0.103	≈0.016 (*µ*_∥_) and ≈0.025 (*µ*_⊥_), at 50% strain	N/A	[75]
P47^b)^	22.8	3.1	≈450	20%	0.088	≈0.0065 (*µ*_∥_) and ≈0.018 (*µ*_⊥_), at 50% strain	N/A	[75]
P48^b)^	21.2	3.0	≈320	20%	0.093	≈0.010 (*µ*_∥_) and ≈0.025 (*µ*_⊥_), at 50% strain	N/A	[75]
P49^b)^	23.2	3.3	≈410	20%	0.064	≈0.0065 (*µ*_∥_) and ≈0.013 (*µ*_⊥_), at 50% strain	N/A	[75]
P50^b)^	18.9	3.9	≈515	40%	0.268	≈0.059 (*µ*_∥_) and ≈0.11 (*µ*_⊥_), at 50% strain	N/A	[75]
P51^b)^	27.2	2.8	≈430	30%	0.288	≈0.032 (*µ*_∥_) and ≈0.060 (*µ*_⊥_), at 50% strain	N/A	[75]
P52^b)^	25.7	3.0	≈390	30%	0.310	≈0.035 (*µ*_∥_) and ≈0.070 (*µ*_⊥_), at 50% strain	N/A	[75]
P53^b)^	20.2	3.1	≈375	20%	0.149	≈0.022 (*µ*_∥_) and ≈0.038 (*µ*_⊥_), at 50% strain	N/A	[75]
P55^b)^	42.4	3.6	700	50%	≈0.38	**≈**0.077 (*µ*_∥_) and ≈0.10 (*µ*_⊥_), at 100% strain	N/A	[74]
P59^b)^	42.4	3.6	330	75%	≈0.48	≈0.11 (*µ*_∥_) and ≈0.051 (*µ*_⊥_), at 100% strain	N/A	[74]
P60^b)^	42.4	3.6	190	>100%	0.65	≈0.50 (*µ*_∥_) and ≈0.21 (*µ*_⊥_), at 100% strain	≈0.12 (*µ*_∥_) and ≈0.07 (*µ*_⊥_), 500 cycles, at 50% strain	[74]
P63^b)^	16.5	4.0	320	100%	0.91	≈0.09 (*µ*_∥_) and ≈0.07 (*µ*_⊥_), at 100% strain	≈0.005 (*µ*_∥_) and ≈0.030 (*µ*_⊥_), 1000 cycles, at 20% strain	[89]
Cross‐linked P63^b)^	16.5	4.0	200	150%	0.40	≈0.15 (*µ*_∥_) and ≈0.13 (*µ*_⊥_), at 100% strain	≈0.03 (*µ*_∥_) and ≈0.08 (*µ*_⊥_), 1000 cycles, at 20% strain	[89]
P65^a)^	305	3.3	430	60%	1.14	0.11 (*µ*_∥_) and 0.09 (*µ*_⊥_), at 100% strain	≈0.4 (*µ*_∥_), 400 cycles, at 60% strain	[88]
P66^a)^	434	1.6	270	>100%	3.24	0.58 (*µ*_∥_) and 0.54 (*µ*_⊥_), at 100% strain	≈2.5 (*µ*_∥_), 400 cycles, at 60% strain	[88]
P67^a)^	244	1.25	450	40%	0.012	0.0098 (*µ*_∥_) and 0.011 (*µ*_⊥_), at 100% strain	0.0024 (*µ*_∥_) and 0.0025 (*µ*_⊥_), 1000 cycles, at 60% strain	[82]
P68^a)^	355	1.51	2090	N/A	0.0087	0.0053 (*µ*_∥_) and 0.0055 (*µ*_⊥_), at 100% strain	0.0056 (*µ*_∥_) and 0.0057 (*µ*_⊥_), 1000 cycles, at 60% strain	[82]
P69^a)^	N/A	N/A	2790	N/A	3.8×10^−5^	7.6×10^−6^ (*µ*_∥_) and 8.0×10^−6^ (*µ*_⊥_), at 100% strain	N/A	[82]
P70^a)^	80	1.42	1380	10%	0.12	0.014 (*µ*_∥_) and 0.017 (*µ*_⊥_), at 100% strain	0.024 (*µ*_∥_) and 0.023 (*µ*_⊥_), 1000 cycles, at 60% strain	[82]
P71^a)^	90	1.30	1200	10%	3.5×10^−4^	2.6×10^−4^ (*µ*_∥_) and 2.4×10^−4^ (*µ*_⊥_), at 100% strain	N/A	[82]
P74^a)^	147.1	2.57	435	50%	0.21	0.10 (*µ*_∥_) and 0.08 (*µ*_⊥_), at 100% strain	N/A	[83]
P75^a)^	201.3	2.34	168	50%	0.13	0.04 (*µ*_∥_) and 0.03 (*µ*_⊥_), at 100% strain	N/A	[83]
P76^a)^	109.7	2.87	72	>100%	0.18	0.21 (*µ*_∥_) and 0.15 (*µ*_⊥_), at 100% strain	0.151 (*µ*_∥_) and 0.075 (*µ*_⊥_), 500 cycles, at 50% strain	[83]
P79^b)^	43.1	2.57	N/A	>100%	≈0.9	≈0.6 (*µ*_∥_) and 0.25 (*µ*_⊥_), at 100% strain	≈0.5 (*µ*_∥_) and 0.7 (*µ*_⊥_), 1000 cycles, at 25% strain	[91]
P80^b)^	51.0	1.74	N/A	40%	0.61	≈0.04 (*µ*_∥_) and ≈0.11 (*µ*_⊥_), at 75% strain	≈0.15 (*µ*_∥_) and ≈0.28 (*µ*_⊥_), 500 cycles, at 25% strain	[90]
P86^b)^	53.6	3.21	N/A	60%	0.82	0.27 (*µ*_∥_) and ≈0.7 (*µ*_⊥_), at 75% strain	0.22 (*µ*_∥_) and 0.38 (*µ*_⊥_), 500 cycles, at 25% strain	[90]
P88^a)^	28.8	1.81	83.7	100%	3.39	3.80 (*µ*_∥_) and 3.14 (*µ*_⊥_), at 150% strain	2.46 (*µ*_∥_) and 1.41 (*µ*_⊥_), 1000 cycles, at 50% strain	[92]
P89^a)^	19.0	1.20	132.9	75%	3.23	1.83 (*µ*_∥_) and 1.35 (*µ*_⊥_), at 150% strain	0.77 (*µ*_∥_) and 0.48 (*µ*_⊥_), 1000 cycles, at 50% strain	[92]
P90^a)^	24.8	1.89	139.6	75%	3.14	1.42 (*µ*_∥_) and 1.27 (*µ*_⊥_), at 150% strain	0.43 (*µ*_∥_) and 0.34 (*µ*_⊥_), 1000 cycles, at 50% strain	[92]
P91^a)^	30.3	1.81	122.2	75%	3.10	1.55 (*µ*_∥_) and 1.62 (*µ*_⊥_), at 150% strain	1.05 (*µ*_∥_) and 0.85 (*µ*_⊥_), 1000 cycles, at 50% strain	[92]

^a)^ Mobilities were obtained from transistors with rigid substrates. The stretched films were transferred to rigid substrates through the transfer‐printing method;^[^ ^94^ ^]^

^b)^ Mobilities were obtained from fully stretchable transistors;

^c)^ The electrical performances of **P38** under strain were evaluated by measuring the conductivity of the doped films.

### 2.1 Incorporation of Conjugation Break Spacers (CBSs) into the Backbones of Polymer Semiconductors

**Scheme 1:**
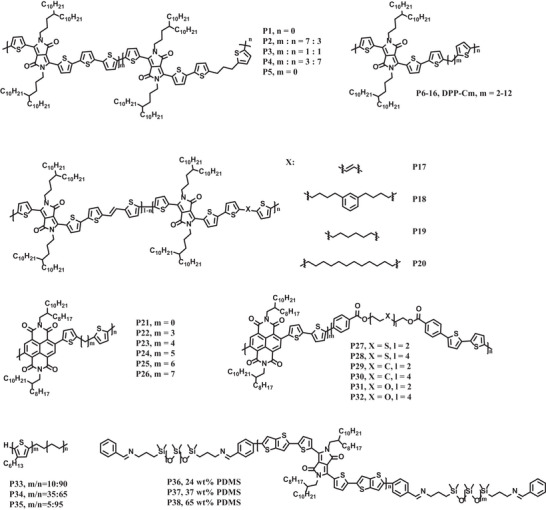
The chemical structures of representative polymer semiconductors incorporating CBSs into the backbones.

Most of polymer semiconductors with high charge mobilities possess planar backbones with minimum torsion and steric hindrance among the conjugated units, leading to the formation of ordered interchain packing and crystalline domains. Consequently, the resulting polymer thin films are brittle under mechanical strain. Rationally breaking the rigidity of the main chain by inserting CBSs into the polymer backbones has proven to be an elegant strategy for endowing polymer semiconductors with the ability to withstand mechanical deformations without producing obvious cracks. The design of these stretchable polymer semiconductors requires a careful balance between the intrinsic charge transporting property and stretchability through the incorporation of the CBSs. In recent years, a wide range of spacer units, including alkyl chains, siloxanes, and other flexible linkers, have been utilized to construct polymer semiconductors with desirable stretchability. The results show that the charge mobilities of the modified polymer semiconductors can be only maintained to a large degree only when the contents of CBSs in the backbones are low, but the elastic modulus, yield strain, and fracture strain can be significantly improved only at high content of CBSs. The chemical structures of representative polymer semiconductors incorporating CBSs into the backbones are presented in **Scheme** **1**.

Mei et al. in 2015 reported a series of DPP‐quaterthiophene copolymers with different contents of ‐CH_2_CH_2_CH_2_‐ units as the flexible CBSs (**P1**–**P5**, Scheme 1).^[^ ^59^ ^]^ They found a remarkable blueshifts in both the solution and thin film absorption spectra from **P1** to **P5** due to the gradual destruction of the conjugation degrees of the backbones. Moreover, hole mobilities decreased gradually in the following order: **P1**>**P2**>**P3**>**P4**>**P5**, by increasing the contents of ‐CH_2_CH_2_CH_2_‐ units. This agreed well with the observation that thin film crystallinity was attenuated for **P1**–**P5** by incorporating more ‐CH_2_CH_2_CH_2_‐ units in their backbones on the basis of Grazing incidence X‐ray diffraction (GIXRD) studies. In addition, **P4** and **P5** exhibited obvious melting transitions ≈270 and 180 °C in differential scanning calorimetry (DSC) analysis, while **P1**–**P3** showed no noticeable thermal transitions. Most interestingly, the hole mobility of the melting‐processed thin film of **P4** reached to 0.30 cm^2^ V^−1^ s^−1^, which was three times as that of the solution‐processed counterpart. Even though they did not study the effect of CBSs on the mechanical property and charge mobility under strain, their pioneer attempts lead to a platform to evaluate the impact of the conjugation break units on the crystallinity of polymer semiconductors in the early stage of this field.

Mei's group also investigated the effect of the length of CBSs on the charge‐transporting property and thin film crystallinity of DPP‐based polymer semiconductors.^[^ ^61^ ^]^ **P6**–**P16** (Scheme 1) are DPP‐based copolymers that contain alkyl spacer units with 2–12 methylenes along the polymer backbones, respectively. They found that the length of alkane spacers had marginal influence on the absorption spectra and energy levels of these polymers. But, the phase transition temperature, the heat of fusion, and surface morphology were profoundly influenced by the length of CBS. Specifically, polymers that incorporated even‐numbered methylene spacers displayed higher melting points than the corresponding adjacent odd‐numbered ones, while the heat of fusion showed an opposite trend. For example, **P11** with seven methylenes as spacer exhibited larger heat of fusion than those of **P10** and P**12** with even‐numbered spacers. In addition, the polymer films with even‐numbered spacers had a more interconnected feature that appeared more fibrillar than the polymers with odd‐numbered spacers. The odd‐even effect was also observed in the decreasing trend of charge mobility by increasing the length of alkane spacers.

**Figure 2:**
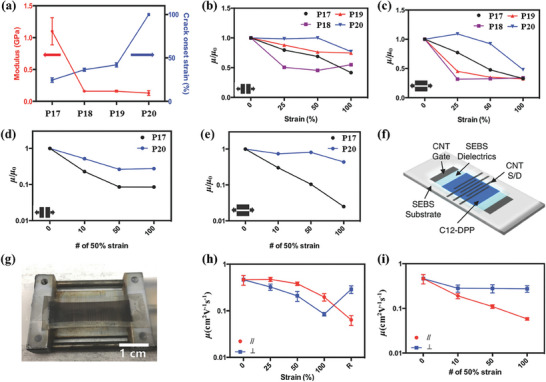
a) Correlation between the flexibility of CBSs and elastic moduli and crack onset strains of the **P17–P20**. b,c) Normalized field‐effect mobilities of the polymer thin films under various strains with charge transporting direction parallel and perpendicular to the strain direction. d,e) Normalized field‐effect mobilities of the polymer thin films after repeated stretching‐releasing cycles with charge transporting direction parallel and perpendicular to the strain direction. f) Illustration of the device architecture of fully stretchable transistors. g) Photograph of the fully stretchable transistor with **P20**. h) Hole mobilities of fully stretchable transistors under various strains with charge transporting direction parallel and perpendicular to the strain direction. i) Hole mobilities of fully stretchable devices after repeated stretching‐releasing cycles with charge transporting direction parallel and perpendicular to the strain direction. Reprinted with permission.^[^ ^62^ ^]^ Copyright 2018, John Wiley and Sons.

Subsequently, Bao et al. systematically investigated the effect of CBSs on both stretchability and charge mobility of polymer semiconductors under different strains.^[^ ^62^ ^]^ Scheme 1 shows the DPP‐based polymers **P18**–**P20** in which three kinds of CBSs with different rigidities and lengths are incorporated into the conjugated chains. They observed that the thin film crystallinity of **P18**–**P20** was reduced, because the intensities of both (100) and (010) peaks of **P18**–**P20** in GIXRD patterns were significantly lower than those of the reference polymer **P17**. Furthermore, the trend of crack onset strains and moduli of these polymers measured by the film‐on elastomer method (**Figure** **2**a) was in a good correlation with the relative degree of crystallinity (rDoC) analysis from GIXRD patterns. In particular, thin film of **P20** showed the lowest modulus of 130 MPa and no obvious cracks could be observed even under 100% strain, indicating that the most flexible CBS showed the strongest ability in regulating the mechanical property of polymer semiconductors. It was also worth mentioning that thin film of **P20** owed more amorphous regions than the other polymers, based on the fact that only **P20** showed *T*_g_ of backbone in dynamic mechanical analysis (DMA). Encouragingly, charge mobilities of organic field‐effect transistors (OFETs) with a top‐contact bottom‐gate architecture of **P18**–**P20** were over 1.00 cm^2^V^−1^s^−1^ on rigid substrates. Even at 100% strain, the charge mobilities of **P20** maintain 77% and 48% of those of the pristine thin films with charge transporting direction parallel and perpendicular to the strain direction, respectively (Figure 2b–e). Finally, in a fully stretchable bottom‐gate‐top‐contact OFET device, **P20** exhibited more than 10% of its initial charge mobility after 100 times of stretching‐releasing cycles at 50% strain (Figure 2f–i). These results imply that incorporation of small contents of CBSs into the backbones of polymer semiconductors is an effective strategy to improve the mechanical property without significantly weakening the charge transporting performance.

Gu and coworkers first reported series of *n*‐type NDI‐based polymers **P21**–**P26** with different lengths of CBS (Scheme 1) in 2020.^[^ ^64^ ^]^ For the first time, Kuhn length (*L*_k_) was employed to quantitatively characterize the flexibility of polymer semiconductors. *L*_k_ was calculated by fitting the respective data of solution small‐angle neutron scattering (SANS). The Kuhn length decreased from 521 Å in **P21** to 36 Å for **P25**. The results showed that both flexibility and crack onset stains of the polymer semiconductors increased with the decrease of the *L*_k_. At the same time, the elastic moduli, glass transition and melting temperatures decreased gradually by increasing the lengths of CBS. GIXRD data also showed that with the increase of CBS lengths, the rDoCs decreased significantly and the original face‐on interchain packing was changed to the edge‐on packing mode.

**Figure 3:**
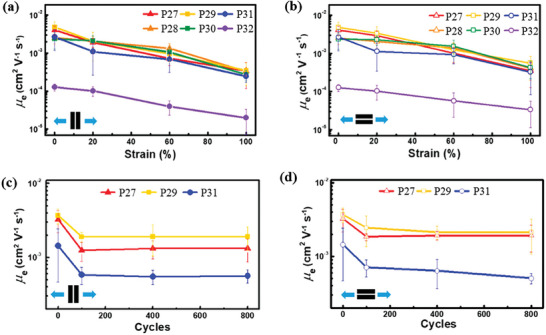
a,b) The electron mobilities of **P27–P32** under different strains with charge transporting direction parallel and perpendicular to the strain direction. c,d) The electron mobilities of **P27**, **P29**, and **P31** after repeated stretching‐releasing cycles with charge transporting direction parallel and perpendicular to the strain direction. Reprinted with permission.^[^ ^58^ ^]^ Copyright 2023, American Chemical Society.

In addition to alkyl spacers, Higashihara, and coworkers reported a new type of NDI‐based polymer with larger CBSs containing S and O heteroatoms very recently.^[^ ^58^ ^]^ **P27**–**P32** (Scheme 1) with 20% contents of CBSs were prepared via Migita–Kosugi–Stille cross‐coupling reaction. The results showed that the introduction of heteroatoms in CBSs causes different effects on the solid‐state packing, intrinsic stretchability, and charge mobility retention under strain for these polymers. For instance, **P31** and **P32** with CBSs containing polar ethylene oxide moieties showed lower crystallinity compared to the other four polymers. This phenomenon could be attributed to the phase separation of ethylene oxide fragments and the conjugated backbones, and thus leading to low crack onset strains and inferior charge mobilities of **P31** and **P32**. In comparison, **P27** and **P28** with CBSs containing thioether moieties displayed better crack onset strains and higher dichroic ratio calculated from the polarized UV‐vis absorption spectra than **P29**–**P32**. Consequently, **P27** and **P28** displayed much higher charge mobilities after both single strain and repeated stretching‐releasing cycles (**Figure** **3**). The superiority of sulfur‐containing CBSs was mainly attributed to the small bond angle of C‐S‐C (98.9°) than C‐O‐C (113.3°) extracted from the optimized configuration of polymer fragments by DFT calculations. The small bond angle of C‐S‐C could lead to a more bent conformation along the polymer backbone, which provided the chain segments of **P21** and **P22** stronger abilities to dissipate strain energy. Taken together, the incorporation of sulfur‐containing CBSs is a powerful molecular engineering approach to offer an elegant balance between mechanical and semiconducting properties.

Polyethylene (PE) was utilized as a special type of CBSs to be incorporated into the backbones of conjugated polymers for developing stretchable polymer semiconductors. **P33**–**P35** (Scheme 1) as diblock polymers of P3HT and polyethylene (PE) were prepared by Müller et al., aiming to explore the influence of the PE moiety on the mechanical and semiconducting properties.^[^ ^45^ ^]^ They showed similar number average molecular weights after controlling the polymerization conditions. The mechanical and electrical properties of the diblock polymers could be tuned by varying the content of PE. With the increase of PE block proportion, the crystallization temperature (*T*_c_) of the P3HT‐fragment gradually decreased, while *T*_c_ of PE‐fragment showed minimal changes. As a result, **P33** displayed a remarkable crack onset stain over 660% and a Young's modulus of 240 MPa, while the homopolymer P3HT cracked at strain of 13%. Encouragingly, **P33** with only 35 wt.% content of P3HT‐fragment showed a hole mobility of 0.05 cm^2^ V^−1^ s^−1^, which was higher than that of the homopolymer P3HT (0.01 cm^2^ V^−1^ s^−1^). The outstanding flexibility and toughness of these diblock copolymers make them hold great potentials for their applications in flexible electronics.

**Figure 4:**
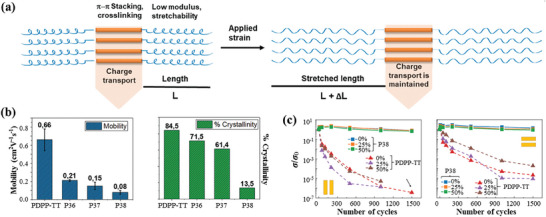
a) Illustration of the design concept and phase separation of **P36–P38**. b) Comparison of charge mobility and crystallinity for PDPP‐TT and **P38**. c) Conductivities of doped thin films of PDPP‐TT and **P38** both under various strains and after repeated stretching‐releasing cycles with charge transporting direction parallel and perpendicular to the strain direction. Reprinted with permission.^[^ ^65^ ^]^ Copyright 2020, John Wiley and Sons.

**P36**–**P38** shown in Scheme 1 are triblock polymers based on poly(diketo‐pyrrolopyrrole‐thienothiophene) (PDPP‐TT), into which different contents of PDMS moieties are introduced.^[^ ^65^ ^]^ PDMS is known to possess many desirable properties such as transparency, low crack onset strain, thermo‐tolerance, resistance to oxidation, ease of fabrication and tunable hardness. The results showed that thin films of **P38** contained more amorphous domains by increasing the contents of PDMS moieties in the backbones (**Figure** **4**a). The relative degree of crystallinity for thin films of PDPP‐TT and **P36**–**P38** decreased in the following order: PDPP‐TT (84.5%) > **P36** (71.5%) > **P37** (61.4%) > **P38** (13.5%) (Figure 4b). Meanwhile, nanophase separation in these triblock polymers occured on the basis of transmission electron microscopy (TEM) analysis. **P38** showed an extremely low elastic modulus of 5 MPa with a crack onset strain of 80%. For the electrical properties, the charge mobility of **P38** reacheed 0.1 cm^2^ V^−1^ s^−1^, which was on the same order of magnitude of the reference polymer PDPP‐TT. Alternatively, the conductivity of the F4‐TCNQ doped films of **P38** was fully maintained after 1500 stretching‐releasing cycles under 50% strain, while the conductivity of doped films of PDPP‐TT decreased dramatically under strain (Figure 4c). Overall, incorporation of PDMS moieties into the backbones of polymer semiconductors is a new way to construct ultrasoft materials with good semiconducting performance.

The results indicate that incorporation of CBSs into the backbones of conjugated polymers is a promising approach for intrinsically stretchable polymer semiconductors. However, significant changes in elastic modulus and the crack onset strain can occur only when the contents of CBSs are high. Unfortunately, polymer semiconductors with more CBSs in the backbones show low charge mobilities in most cases. Further optimizations of CBSs structures, as well as the in‐depth understanding of relationship between the structures of CBSs and the evolution of aggregate structures of polymer chains under strain, are still needed.

### 2.2 Incorporation of Dynamic Non‐Covalent Bonding Units

It is known that dynamic non‐covalent bonds can selectively undergo reversible breaking and reformation upon external stimuli including mechanical stress and heating. Recent results demonstrate that incorporation of dynamic non‐covalent bonding units into polymer semiconductors is beneficial for not only promoting the interchain packing order and boosting charge mobility, but also improving the stretchability. This is achieved by using dynamic non‐covalent bonds as non‐covalent cross‐linking sites to dissipate strain energy. Specifically, when the thin film of polymer semiconductor is subjected to strain, the dynamic bonds can be broken, and the mechanical stress is redistributed throughout the thin film without producing cracks. As a result, the polymer semiconductor can withstand large deformations without losing the semiconducting performance.

**Scheme 2:**
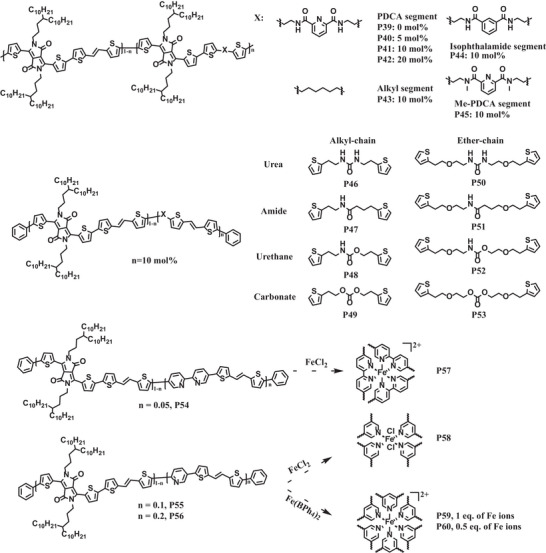
The chemical structures of representative polymer semiconductors incorporating dynamic non‐covalent bonding units.

As typical dynamic non‐covalent bonding moieties, hydrogen bonding, and metal–ligand coordination units have been introduced into polymer semiconductors via either side‐chain engineering or main‐chain modification for developing stretchable polymer semiconductors. The chemical structures of representative polymer semiconductors incorporating dynamic non‐covalent bonding units are presented in **Scheme** **2**.

**Figure 5:**
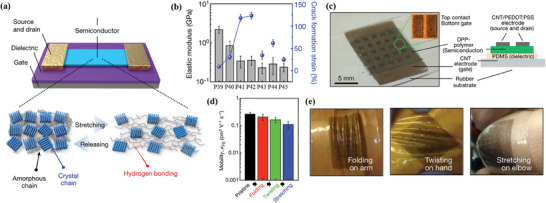
a) Illustration of the mechanism for dynamic bonding induced enhancement of stretchability. (b) Elastic moduli and crack onset strains of **P39–P45**. c) Photograph and device configuration of the flexible transistor array with **P42**. d) Charge mobilities of the polymer thin films of **P42** in bending, twisting, and stretching state. e) Photographs of the FET devices with **P42** on skin. Reprinted with permission.^[^ ^76^ ^]^ Copyright 2016, Springer Nature.

In 2016, Bao et al. designed and synthesized polymers **P40**–**P42** (Scheme 2),^[^ ^76^ ^]^ by incorporating 2,6‐pyridine dicarboxamide (PDCA) groups as hydrogen bonding units into the main chains of DPP‐based conjugated polymers. For comparison, **P39** without PDCA units and **P43** with alkyl chain segment were also prepared. Despite the hydrogen bonding network formed in the polymers, the elastic moduli decreased significantly by increasing the content of PDCA units (**Figure** **5**a,b). This was mainly due to the fact that PDCA could break the rigid structure of the polymer skeleton and enhance the proportion of amorphous domains, on the basis of the GIXRD data. Consequently, the crack onset stains increased significantly as the proportion of PDCA increases. For instance, microscale cracks on thin film of **P42** were only formed when strain was higher than 120%. Compared with reference polymer **P43** containing alkyl segment and **P45** containing Me‐PDCA, the stretchability of **P41** was significantly improved mainly due to the introduction of hydrogen bonds. Moreover, the elastic modulus of **P41** is much higher than that of **P44**, suggesting that the introduction of pyridine groups might improve mechanical properties by participating in forming intramolecular and intermolecular hydrogen bonding. As for the electrical properties, charge mobilities of **P40**–**P42** decreased slightly as the proportion of PDCA increased. When the content of PDCA reached 20%, the charge mobility of **P42** was still up to 0.58 cm^2^ V^−1^ s^−1^. Remarkably, the charge mobility of **P42** was kept ≈0.1 cm^2^ V^−1^ s^−1^ after bending, twisting, and stretching (Figure 5c–e). The results demonstrate that the incorporation of H‐bonding units into the main chains of conjugated polymers is an efficient strategy to construct stretchable polymer semiconductors.

To further systematically investigate the effect of the flexibility and the strength of the hydrogen bonding interaction on the mechanical and semiconducting performance of conjugated polymers, Bao and coworkers synthesized eight DPP‐based polymers (**P46**–**P53**, Scheme 2).^[^ ^75^ ^]^ Urea, amide, urethane, and carbonate groups with similar structures were introduced into the backbones of DPP‐based polymers and served as fair comparisons as the precursor units of hydrogen bonding in these polymers. The π‐conjugated backbones and the hydrogen bonding units and the analogues were linked by either alkyl or ether groups to provide different flexibility. **P52** and **P53** with carbonate groups that can not yield hydrogen bonding were also prepared for comparison. The hydrogen bonding strength within these polymers decreases in the following order: urea > amide > urethane. Expectedly, **P47** containing ether and urea groups exhibited the highest crack onset strain among these polymers. In comparison, both **P52** and **P53** showed low crack onset strains as the fully conjugated polymers without hydrogen bonding units. As for the hole mobilities of thin films of **P47**, **P49**, **P51,** and **P53** under various strains, the degradation along the stretching direction was consistent with the trend of crack onset strain. This work demonstrates the essential roles of the flexibility of hydrogen bonding units and the strength of the hydrogen bonding interaction on the performances of stretchable polymer semiconductors.

In addition to hydrogen bonds, metal‐ligand coordination bonds can also be used as dissipative elements of strain energy. In 2021, Bao et al. reported that the ductility of pyridine‐containing DPP‐based polymers could be tuned by the coordination of metal ions with pyridine units (**P54**–**P60**, Scheme 2).^[^ ^74^ ^]^ It was found that the elastic moduli of **P54**, **P55**, and **P56** decreased significantly compared to the reference polymer PDPPTVT without pyridine units, because the introduction of ligands could partially disturb the interchain packing order. Moreover, the elastic moduli of polymers that were complexed with iron ions were decreased further due to the coordination‐induced disorder. Specifically, the cross‐linking of multiple polymer chains originated from the metal‐ligand coordination could potentially reduce the overall thin film crystallinity. The results showed that the elastic modulus of **P58** with weaker coordination bonds was lower than that of **P57** with strong coordination bonds. Similarly, the elastic modulus of **P59** was lower than that of **P58**. The insertion of ligands also led to a slightly decrease of charge mobility for **P55** compared to the reference polymer PDPPTVT. However, a surprisingly high charge mobility of 2.2 cm^2^ V^−1^s^−1^ was obtained for **P60** with partially coordination bonds due to the doping effect of iron ions. Charge mobilities of the polymer thin films of **P55**, **P59,** and **P60** under various strains with charge transporting direction parallel to the strain direction were also evaluated. Among **P55**, **P59,** and **P60**, **P60** with moderate coordination density exhibited the least variation of charge mobilities under different strains. Altogether, incorporation of dynamic metal‐ligand coordination bonding units into the backbones of polymer semiconductors is able to boost both charge mobility and stretchability simultaneously.

The incorporation of dynamic bonding units into polymer semiconductors has been proved to be an innovative strategy to generate stretchable polymers with high mobilities. Precise control of the cross‐linking degree caused by the dynamic bonding interactions is crucial for regulation of mechanical properties of polymer semiconductors. Encouragingly, metal‐ligand coordination units can not only be used as stress dissipation sites under external forces, but also act as dopants to enhance charge mobility. It is worthwhile to further expand this strategy for future molecular design for high performance stretchable polymer semiconductors.

### 2.3 Incorporation of Flexible Side Chains

**Scheme 3:**
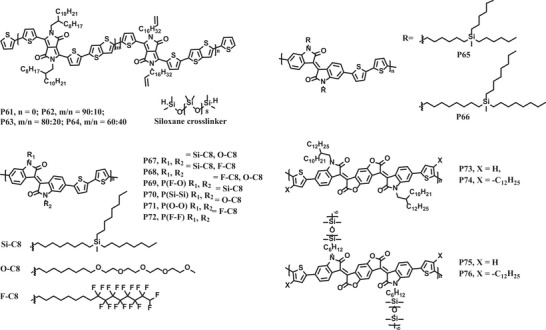
The chemical structures of representative polymer semiconductors incorporating flexible side chains.

Unlike most of the approaches discussed above to improve the stretchability of polymer semiconductors involving main chain modification, side chain engineering is an elegant strategy that will not break the conjugation of the backbone. In the past few years, many pioneering efforts have focused on introducing flexible side chains into polymer semiconductors.^[^ ^56^, ^57^, ^79^, ^80^, ^81^, ^82^, ^83^, ^84^, ^85^, ^86^, ^87^, ^88^, ^89^ ^]^ These flexible side chains include but are not limited to siloxane, carbosilane, oligoether, poly(butyl acrylate), and semifluorinated side chains. The hydrophilicity, symmetry, and distribution of these flexible side chains also have significant influence on the distribution of crystalline domains and amorphous regions in conjugated polymer films. In the following section, we will present some representative developments about stretchable polymer semiconductors grafted with flexible side chains. The chemical structures of representative polymer semiconductors incorporating flexible side chains are shown in **Scheme** **3**.

Siloxane is a kind of flexible chain because the Si‐O‐Si bonds can easily deform over a large range of angle change. Bao et al. designed and synthesized a series of DPP‐based copolymers with different ratios of alkene‐terminated side‐chains (**P61**–**P64**, Scheme 3).^[^ ^89^ ^]^ Then, the siloxane oligomers were introduced into polymers by post‐polymerization modification, which involved the crosslinking of alkenes and siloxane oligomers though hydrosilylation in the presence of Karstedt's catalyst. As expected, the flexible siloxane could hinder the rearrangement and packing of conjugated backbones, and as a result the cross‐linked polymers showed weaker overall diffraction intensities in GIXRD patterns than those of the pristine one. Accordingly, the tensile modulus of the cross‐linked **P63** was significantly lower than that of **P63**, indicating that the ductility of the cross‐linked **P63** was improved. Although the crosslinked thin film of **P63** exhibited a relatively low initial average mobility of 0.66 cm^2^ V^−1^ s^−1^, a high mobility of 0.40 cm^2^ V^−1^ s^−1^ could still be maintained after 500 stretching‐releasing cycles under 20% strain with charge transporting direction perpendicular to the strain direction.

Chen and coworkers have explored side‐chain engineering for polymer semiconductors in recent years.^[^ ^82^, ^84^, ^85^, ^86^, ^88^ ^]^ In 2016, they synthesized two IID‐based conjugated polymers with long and branched carbosilane side chains (**P65** and **P66**, Scheme 3).^[^ ^88^ ^]^ When the branching point of side chains was moved away from the main chains, the packing order and charge transport performances were improved. A high hole mobility of 8.06 cm^2^ V^−1^ s^−1^ was obtained for **P66**. Due to the excellent flexibility of carbosilane side chains, **P65** and **P66** exhibited low elastic moduli and superior thin film ductility. The charge mobility of **P66** was maintained over 1 cm^2^ V^−1^ s^−1^ at 60% strain after 400 stretching‐releasing cycles with charge transporting direction parallel and perpendicular to the strain direction. In addition to carbosilane, they also found that incorporation of other types of side chains such as oligoether and semifluorinated alky chains into IID‐based polymers (**P67** and **P72**, Scheme 3) also led to similar effects on the mechanical and electrical properties.^[^ ^82^ ^]^

To date, only few stretchable *n*‐type conjugated polymers have been reported due to the low electron mobility under high strain. The exploration of stretchable *n*‐type polymers with high mobility is of great importance for the development of organic flexible electronic devices. In 2021, Qiu et al. reported four bis(2‐oxoindolin‐3‐ylidene)‐benzodifuran‐dione (BIBDF)‐based conjugated polymers with different side chains (**P73**–**P76**, Scheme 3).^[^ ^83^ ^]^ The effects of side chain type and graft density on the mechanical properties of these polymers were investigated. The results showed that the polymer crystallinity could be designedly reduced by replacing branched alkyl side chains with linear hybrid siloxane‐based side chains and increasing the side chain density. Specifically, compared to thin films of **P73**–**P75**, thin film of **P76** possessed shorter coherence lengths (CLs) and smaller rDoC on the basis of the respective GIXRD patterns. The elastic modulus and crack onset strain of **P76** were significantly improved. It was worth noting that the linear siloxane‐terminated side chains had little effect on the electrical properties and **P74**–**P76** showed similar electron mobilities ≈0.9 cm^2^ V^−1^ s^−1^. But, **P76** showed a higher electron mobility than the initial thin film under 100% strain in the direction parallel to the strain due to the stretch‐induced chain alignment. For the long‐term stretchability, 72% and 50% of the initial electron mobilities were maintained for **P76**, respectively, in the parallel and perpendicular direction under 50% strain after 500 stretching‐releasing cycles. The studies demonstrate that the stretchability and electron mobilities of polymer semiconductors can be simultaneously improved by precise selection of the types and distribution of flexible side chains.

We have summarized recent progresses about the incorporation of flexible side chains into conjugated polymers to enhance the stretchability of polymer semiconductors. By carefully selecting the flexible side chains, the thin film crystallinity, tensile moduli, and crack onset strains of these polymers can be significantly altered. However, in most cases, the large volume ratio of side chains can lower charge mobility. Further optimization of the chemical structure of side chains is still required to achieve a trade‐off between stretchability and charge‐transporting properties.

### 2.4 Random Copolymerization

**Scheme 4:**
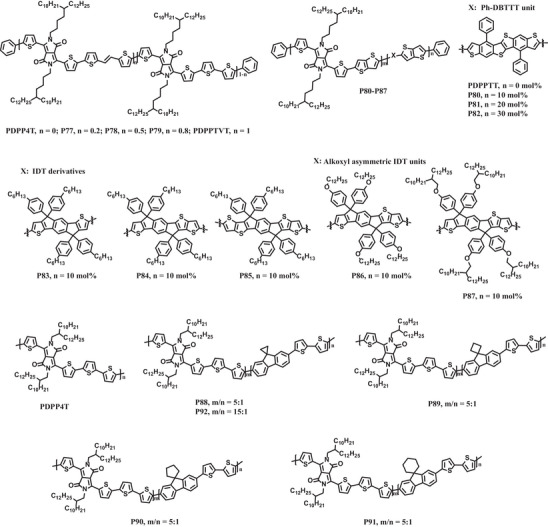
The chemical structures of representative stretchable polymer semiconductors prepared by random copolymerization.

As mentioned above, most of the strategies to improve stretchability of polymer semiconductors inevitably compromise the electrical performance. Random copolymerization is considered to be a simple and effective approach to address this issue. The specific design principle involves reducing the overall crystallinity of polymer semiconductors while maintaining or even enhancing the short‐range ordered aggregates by randomly inserting different conjugated units into the conjugated backbones. As a result, the distribution of hard crystalline domains and soft amorphous domains can be optimized. The advantage of this strategy is that it involves neither breaking of the conjugation degree of the main chain nor introduction of large bulky side chains. The chemical structures of representative stretchable polymer semiconductors prepared by random copolymerization are presented in **Scheme** **4**.

**Figure 6:**
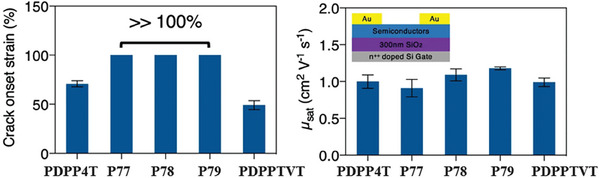
The crack onset strains and hole mobilities of PDPPTVT, PDPP4T, and **P77–P79**. Reprinted with permission.^[^ ^91^ ^]^ Copyright 2021, Springer Nature.

In 2021, Bao's group reported terpolymer‐based stretchable semiconductors comprising DPP units and different molar ratios of thienylenevinylene (TVT) units and bithiophene (BT) units (**P77**‐**P79**, Scheme 4).^[^ ^91^ ^]^ The GIXRD results indicated that the rDoCs of these terpolymers were lower than those of the regular copolymers. All terpolymers exhibited improved crack onset strains (>100%) compared to both the regular copolymers with only one type of donor units and the blends of the reference polymer PDPPTVT and PDPP4T (**Figure** **6**). Moreover, the mechanical reversibility of these terpolymers was also enhanced. There were no obvious wrinkles in the thin film of **P79** even after 500 stretching‐releasing cycles at 25% strain. Importantly, the charge mobility of **P79** could be largely maintained under repeated strains (25%) of 1000 cycles. Later, they also extended this strategy to other conjugated polymer systems (**P80**–**P87**, Scheme 4).^[^ ^90^ ^]^ These results manifest that random copolymerization is a general approach to achieve stretchable semiconducting polymers with high charge carrier mobilities.

**Figure 7:**
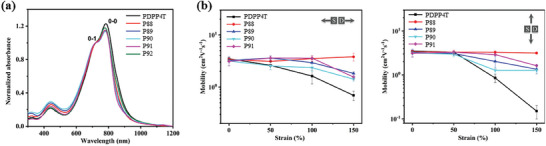
a) Absorption spectra of the reference polymer PDPP4T and the terpolymers **P88–P92**. b) Charge mobilities of the reference polymer PDPP4T and the terpolymers **P88–P91** under various strains with charge transporting direction parallel and perpendicular to the strain direction. Reprinted with permission.^[^ ^92^ ^]^ Copyright 2023, John Wiley and Sons.

Some of us have recently reported a series of terpolymers by incorporating non‐centrosymmetric spiro‐fluorene units attached with various cycloalkane rings into the main chain of DPP‐based conjugated polymers (**P88**‐**P92**, Scheme 4).^[^ ^92^ ^]^ All terpolymers showed similar UV‐vis‐NIR absorption spectra, but their absorption intensity ratios *I*_0‐0_/*I*_0‐1_ were slightly lower than that of parent polymer, suggesting that the introduction of spiro‐fluorene units moderately disrupted the linear configuration of the main chain and reduced the interchain stacking degree (**Figure** **7**a). GIXRD results also confirmed that the terpolymers **P88**‐**P92** possessed lower rDoC. Interestingly, the interchain *π–π* stacking distance was shortened from 3.85 Å in parent polymer to ≈3.76 Å in terpolymers due to the reduced content of steric hindrance bulky alkyl chains linked to DPP units in the terpolymers. These results indicated that these terpolymers could form small crystalline domains, a relatively ideal morphology for efficient charge transporting and good stretchability. Consequently, all terpolymers showed higher crack onset strains and lower tensile moduli than the parent polymer. Among them, **P88** containing spiro[cyclpropane‐1,9′‐fluorene] exhibited the best mechanical properties with a crack onset strain >75% and a tensile modulus of 83.7 MPa. Furthermore, this terpolymer showed charge mobility as high as 3.1 cm^2^ V^−1^ s^−1^ at even 150% strain and 1.4 cm^2^ V^−1^ s^−1^ after 1000 stretching‐releasing cycles at 50% strain (Figure 7b). This study highlights the importance of carefully selecting terpolymeric units for developing intrinsically stretchable polymer semiconductors with high charge mobilities.

## 3 Healable Polymer Semiconductors

**Figure 8:**
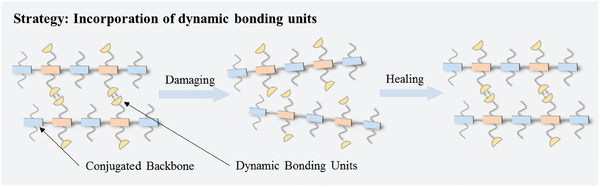
Illustration of the molecular design strategy for healable polymer semiconductors.

**Scheme 5:**
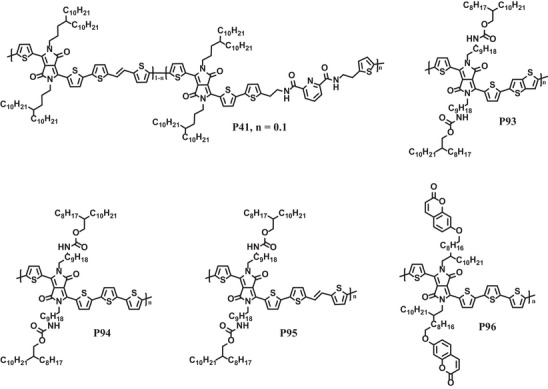
The chemical structures of representative healable polymer semiconductors.

Although a variety of the reported intrinsically stretchable polymer semiconductors can maintain their electrical performances under strains, in most cases the charge transporting performances are significantly degraded during multiple strain‐releasing cycles. In fact, flexible electronic devices in practical application environments require polymer semiconductors to withstand repeated deformations over long‐term usage. For example, the flexible health monitoring device attached to human heart needs to undergo ≈100 000 deformations per day. Traditional polymer semiconductors are difficult to repair after being damaged or degraded. In contrast, healable polymer semiconductors offer a promising solution by allowing the restoration of functions after healable processes. In this regard, some pioneering efforts have been focused on the design and synthesis of polymer semiconductors with healing ability. Specifically, two strategies have been explored for healable polymer semiconductors. These include i) blending a polymer semiconductor with a healable elastomer can integrate the healable feature with the semiconducting and stretchable properties,^[^ ^77^, ^95^ ^]^ and ii) incorporation of dynamic bonding units is also feasible to provide polymer semiconductor driving force to heal cracks after continuous mechanical deformation (**Figure** **8**).^[^ ^78^, ^96^, ^97^ ^]^ The first strategy involves multiple components and complex device fabrication processes. We will mainly discuss the strategy with dynamic bonding units for the development of healable polymer semiconductors. **Table** **2** summarizes the variation of charge mobilities before and after healing processes for healable polymer semiconductors. The chemical structures of representative healable polymer semiconductors are shown in **Scheme** **5**.

**Table 2:** Structural and performance parameters of healable polymer semiconductors.

Polymer	*M*n [kDa]	PDI	Initial mobility [cm^2^ V^−1^ s^−1^]	Mobility after healing [cm^2^ V^−1^ s^−1^]	Healing conditions	Reference
P41	16.2	3.0	1.28	1.13	Solvent vapor and heat	[76]
P93	154	1.15	1.75×10^−2^	≈1.79×10^−2^	Solvent vapor and heat	[78]
P94	164	1.12	2.69×10^−2^	≈0.94×10^−2^	Solvent vapor and heat	[78]
P95	171	1.10	3.04×10^−2^	≈1.22×10^−2^	Solvent vapor and heat	[78]
P96	33.8	1.26	4.96×10^−3^	4.98×10^−3^	Solvent vapor, UV irradiation, and heat	[97]

**Figure 9:**
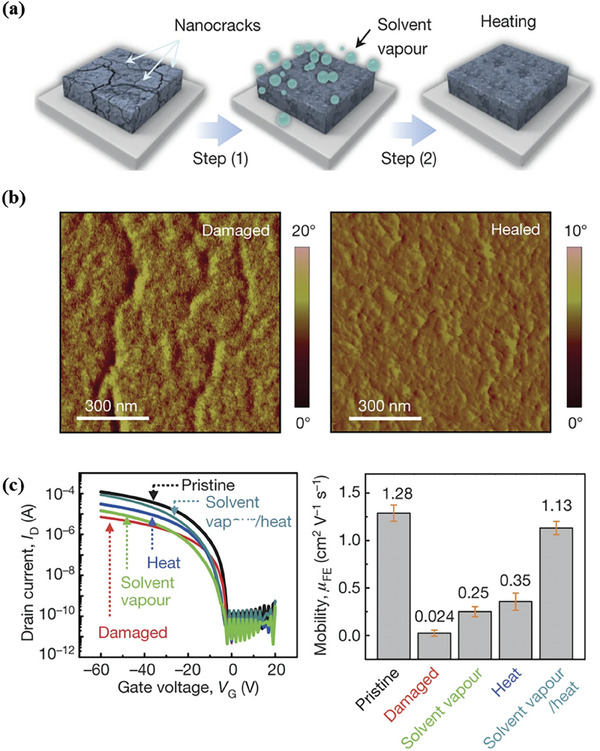
a) Illustration of the healing process. b) AFM images of the damaged and healed films of **P41**. c) Transfer curves, and charge mobilities of **P3** before and after different healing processes. Reprinted with permission.^[^ ^76^ ^]^ Copyright 2016, Springer Nature.

The incorporation of dynamic bonding units into polymer semiconductors can not only improve the stretchability by utilizing dynamic bonding networks as strain energy dissipation sites, but also endow polymer semiconductor with healing ability by using the reversible breaking and reformation features under specific circumstances to repair damages caused by mechanical deformations. In 2016, Bao et al. reported a highly stretchable and healable polymer semiconductor incorporating 2,6‐pyridine dicarboxamide (PDCA) units in the main chain as the dynamic hydrogen bonds (**P41**, Scheme 5).^[^ ^96^ ^]^ Due to the highly dynamic reversibility of the hydrogen bonds, the damaged **P41** films could be quickly reconstructed with post treatments (**Figure** **9**a). The size and density of the nanocracks in damaged **P41** films could be greatly repaired after combination of solvent vapor and thermal annealing (Figure 9b). More importantly, charge mobility of the healed film can be recovered to 1.13 cm^2^ V^−1^ s^−1^ with a healing efficiency of 88% (Figure 9c). It is also feasible that healable polymer semiconductors can be constructed by incorporating dynamic bonding units in the side chains. Oh and coworkers synthesized three DPP‐based copolymers with urethane‐containing side chains (**P93**‐**P95**, Scheme 5).^[^ ^78^ ^]^ The moderate hydrogen bonding strength among urethane units facilitate the molecular motion and hydrogen bond recombination during the healing process.

**Figure 10:**
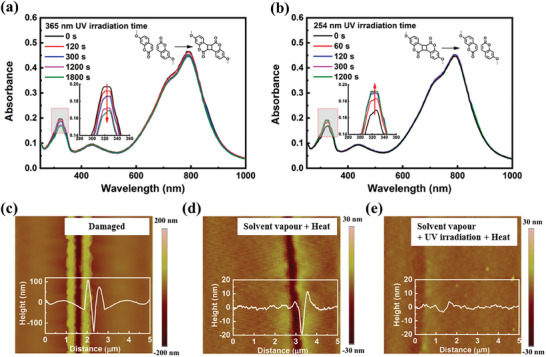
a) Thin‐film absorption spectra of **P96** exposed to 365 nm UV irradiation for different times. b) Thin‐film absorption spectra of **P96** exposed to 365 nm UV irradiation for 1800 s and then 254 nm irradiation for different times. c–e) AFM height images of the damaged film and the healed thin film of **P96**. Reprinted with permission.^[^ ^97^ ^]^ Copyright 2022, John Wiley and Sons.

In addition to dynamic hydrogen bonds, the incorporation of dynamic covalent bonding units can also integrate unique healing property with polymer semiconductors. Some of us have reported a DPP‐based polymer containing coumarin groups at the end of side chains (**P96**, Scheme 5).^[^ ^97^ ^]^ As evidenced by the absorption spectra, the photo‐crosslinked dimer of coumarin groups could be reversibly dissociated into the corresponding monomer after exposure to 254 nm light (**Figure** **10**a,b). In order to verify the effectiveness of coumarin groups in constructing healable organic semiconductors, the authors used AFM probe to completely cut the channel of OFET device, and then examined the variation of the charge mobility of **P96** before and after healing process (Figure 10c–e). It was proved that the photo‐crosslinking of coumarin units could significantly promote the healing behavior for the damaged thin film. The healing efficiency was more than 90% in terms of the restoration of charge mobility after the post‐treatments combining solvent vapor, ultraviolet illumination and thermal annealing. They also found that the photo‐crosslinked thin film of **P96** exhibits good thermal stability with regard to the charge transporting performance.

Overall, healable polymer semiconductors are less explored. In this section, we present a few healable polymer semiconductors by incorporation of dynamic bonding units into either backbones or side chains. However, these healable polymer semiconductors exhibit low charge mobilities and the healing process can only occur after being triggered by external stimuli such as solvent vapor, heating or light irradiation. New molecular design strategies are urgently needed for self‐healable polymer semiconductors, wherein the healing process after mechanical deformation can occur automatically under mild conditions.

## 4 Summary and Outlook

In this review, we discuss molecular design strategies for intrinsically stretchable and healable polymer semiconductors, and intend to shed light on how the chemical structure impacts the morphology evolution under stress and during the healing process. There is a rich and versatile toolbox to endow the conventional polymer semiconductors with desirable mechanical properties without significantly compromising the semiconducting performance. These strategies include the incorporation of conjugation break spacers, dynamic bonding units, flexible side chains, and third copolymerization components into the polymer semiconductors, reducing the overall crystallinity of conjugated polymers and create more stress dissipation areas. Such approaches contribute to the continuous emergence of new polymer semiconductors with extraordinary durability. Beyond the breakthrough in mechanical performance improvements, thrilling results about charge mobilities both under strain and after repeated stretching‐releasing cycles are also achieved. Polymer semiconductors with charge mobility over 1 cm^2^ V^−1^ s^−1^ after 1000 stretching‐releasing cycles at 50% strain are obtained.^[^ ^92^ ^]^ Additionally, the incorporation of dynamic bonding units into backbones and side chains of conjugated polymers can afford healable polymer semiconductors. It is expected that the excellent repair efficiency of healable polymer semiconductors can prolong the lifespan of electronic devices.

Despite these encouraging progresses, this interdisciplinary area is at the early stage and further investigations are needed. First, polymer semiconductors with both high charge mobility and extraordinary durability remain limited. Although intensive efforts have been focused on improving the stretchability of polymer semiconductors without compromising the electrical performance in recent years, charge mobilities of most stretchable polymer semiconductors are below 1 cm^2^ V^−1^ s^−1^. Fundamentally, achieving a delicate balance between the mutually contradictory demands of charge mobility and stretchability on aggregate structures is still very challenging for polymer semiconductors, and new elegant design strategy is highly demanding. Second, stretchable *n*‐type and bipolar polymer semiconductors have received less attention. The majority of the reported stretchable polymer semiconductors are based on *p*‐type conjugated polymers. It is expected that similar strategies can be applied to *n*‐type and bipolar conjugated polymers, yielding stretchable *n*‐type and bipolar polymer semiconductors. Third, more studies are warranted for polymer semiconductors with a high elastic range. Currently, the crack onset strains of many stretchable polymer semiconductors have already exceeded 100%. However, most deformations at high strains are plastic, meaning that thin films can not return to their original shape when the stress is removed. The irreversible morphology evolution beyond the elastic range is the main reason for mobility degradation after multiple stretching‐releasing cycles. Fourth, innovative strategies are needed for self‐healable polymer semiconductors. Post‐treatments, which involve the combination of multiple external stimuli, are required to restore the performances of the reported healable polymer semiconductors. In comparison, self‐healable polymer semiconductors, for which the healing process can occur automatically under mild conditions, will hold promise for stretchable electronic devices with improved functionality and longevity.

Overall, the primary consideration in molecular design for stretchable and healable polymer semiconductors is how to achieve an ideal thin film morphology that can maintain high‐quality continuous charge transporting channels during the stress dissipation process. New molecular engineering approaches and in situ characterization methods are essential for further integration of mechanical and electrical properties. Moreover, revealing the in‐depth morphology evolution under strain or during the healable process will further pave the way for creating high‐performance stretchable and healable polymer semiconductors.

## Conflict of Interest

The authors declare no conflict of interest.
